# TME-NET: an interpretable deep neural network for predicting pan-cancer immune checkpoint inhibitor responses

**DOI:** 10.1093/bib/bbae410

**Published:** 2024-08-21

**Authors:** Xiaobao Ding, Lin Zhang, Ming Fan, Lihua Li

**Affiliations:** Institute of Biomedical Engineering and Instrumentation, Hangzhou Dianzi University, Hangzhou 310018, Zhejiang, China; Institute of Big Data and Artificial Intelligence in Medicine, School of Electronics and Information Engineering, Taizhou University, Taizhou 318000, Zhejiang, China; School of Computer Science and Technology, Hangzhou Dianzi University, Hangzhou, 310018, China; Institute of Biomedical Engineering and Instrumentation, Hangzhou Dianzi University, Hangzhou 310018, Zhejiang, China; Institute of Biomedical Engineering and Instrumentation, Hangzhou Dianzi University, Hangzhou 310018, Zhejiang, China; Institute of Biomedical Engineering and Instrumentation, Hangzhou Dianzi University, Hangzhou 310018, Zhejiang, China; School of Computer Science and Technology, Hangzhou Dianzi University, Hangzhou, 310018, China

**Keywords:** immune checkpoint inhibitor, immunotherapy, tumor microenvironment, deep learning

## Abstract

Immunotherapy with immune checkpoint inhibitors (ICIs) is increasingly used to treat various tumor types. Determining patient responses to ICIs presents a significant clinical challenge. Although components of the tumor microenvironment (TME) are used to predict patient outcomes, comprehensive assessments of the TME are frequently overlooked. Using a top-down approach, the TME was divided into five layers—outcome, immune role, cell, cellular component, and gene. Using this structure, a neural network called TME-NET was developed to predict responses to ICIs. Model parameter weights and cell ablation studies were used to investigate the influence of TME components. The model was developed and evaluated using a pan-cancer cohort of 948 patients across four cancer types, with Area Under the Curve (AUC) and accuracy as performance metrics. Results show that TME-NET surpasses established models such as support vector machine and k-nearest neighbors in AUC and accuracy. Visualization of model parameter weights showed that at the cellular layer, Th1 cells enhance immune responses, whereas myeloid-derived suppressor cells and M2 macrophages show strong immunosuppressive effects. Cell ablation studies further confirmed the impact of these cells. At the gene layer, the transcription factors STAT4 in Th1 cells and IRF4 in M2 macrophages significantly affect TME dynamics. Additionally, the cytokine-encoding genes IFNG from Th1 cells and ARG1 from M2 macrophages are crucial for modulating immune responses within the TME. Survival data from immunotherapy cohorts confirmed the prognostic ability of these markers, with *p*-values <0.01. In summary, TME-NET performs well in predicting immunotherapy responses and offers interpretable insights into the immunotherapy process. It can be customized at https://immbal.shinyapps.io/TME-NET.

## Graphical Abstract

**Graphical Abstract:**
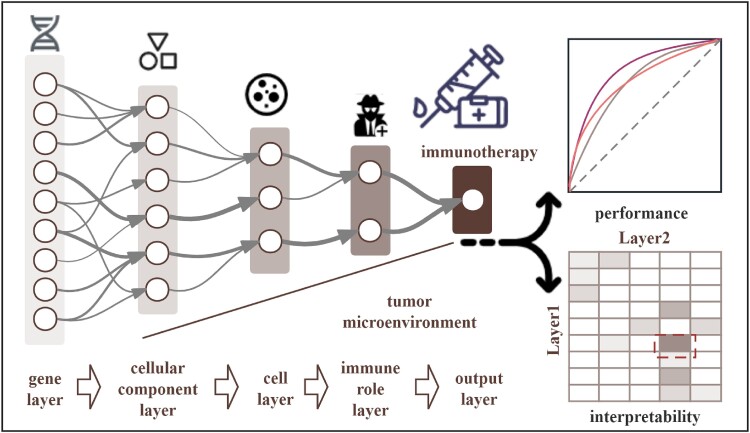

## Introduction

Recent advances in oncology have been significantly propelled by the development of immune checkpoint inhibitors (ICIs), ushering in a new era of cancer treatment. These agents specifically target pivotal regulatory proteins such as PD-1, PD-L1, and CTLA-4, which are crucial for modulating immune responses [1–3]. By inhibiting signals that suppress immune defenses, ICIs enable T cells to detect and attack cancer cells [4, 5]. Although ICIs are applied across a broad spectrum of tumor types, their efficacy is limited to a subset of patients. Moreover, the potential for adverse effects necessitates careful consideration, posing a critical and persistent challenge in determining which patients are likely to benefit from ICI therapy.

To address this challenge, the identification of effective biomarkers is a critical strategy. In clinical settings, tumor mutational burden (TMB) [6], PD-1 [7], and PD-L1 [8] are commonly used as biomarkers to predict the outcome of immunotherapy. However, given the complexity and dynamic nature of tumor immunity, these biomarkers are not always effective [9–11]. To enhance the accuracy of predicting responses to immunotherapy, it is essential to explore a more diverse array of biomarkers that can better represent the heterogeneity of treatment outcomes. Accumulating evidence highlights the critical role of the tumor microenvironment (TME) in tumor immunity, particularly through its regulation of immune cell infiltration and immune evasion, which significantly affects the efficacy of ICIs [12, 13]. Studies have demonstrated that understanding the components and component interactions within the TME can guide the identification and utilization of novel biomarkers to optimize treatment outcomes [14, 15]. Researchers have conducted in-depth analyses of the TME from multiple perspectives, including the quantification of immune cells [16], pathway activity [17, 18], transcription factor activity [19], ligand-receptor pairs [20], and cell–cell pairs [21, 22]. These efforts have deepened our understanding of the TME, revealing numerous potential biomarkers for immunotherapy. However, the complexity of tumor immune behavior cannot be fully understood or captured by any single, reductionist approach to the TME. This underscores the significant gap in accurately predicting responses to immunotherapy, emphasizing the need for a holistic strategy to enhance prediction accuracy.

Integrating multiple biomarkers into machine learning models represents a holistic approach. The established TME biomarkers offer a rich array of features for machine learning models. To further enhance the accuracy and efficacy of these models, extensive prior knowledge is incorporated to refine the feature set. These include protein–protein interactions (PPI), pathway [23, 24], and cell markers obtained through single-cell sequencing techniques [25]. Compared to using a single TME biomarker, machine learning models, particularly deep learning models, can achieve greater accuracy and efficacy. However, these methods often lack interpretability and sometimes fail to provide crucial insights into the TME, which is essential for fully understanding immune responses.

To strike a balance between performance and interpretability, a framework that integrates both aspects is crucial for prediction models. Recently, the development of an interpretable model guided by biological insights has made significant advancements. Computational models such as P-NET [26] and PASNet [27] classify patients and predict outcomes using biological data. DCell [28] and its successor, DrugCell [29], simulate cellular behaviors and optimize drug regimens by targeting specific molecular pathways. GenNet [30] leverages deep learning to predict disease phenotypes from genetic variants, enhancing the precision of treatment strategies. This body of research highlights the critical role of incorporating biological frameworks into prediction models. The successful application of such models, which leverage prior biological knowledge, implies that the TME serves as an essential foundation for developing interpretable immunotherapy prediction models.

Tumor immunity, a critical aspect of the TME, constitutes a complex and intricately regulated system, encompassing a variety of immune cell subtypes. These subtypes play distinct roles within the TME, including cells that combat tumors and those that promote tumor progression, collectively contributing to and shaping the TME [31]. Immuno-oncology research has extensively explored the characteristics and functions of these two cell subtypes. Within the anti-tumor subtype, key players include Th1 cells, CD8+ T cells, dendritic cells (DC), M1-type macrophages, and natural killer (NK) cells. On the other hand, the pro-tumor subtype primarily consists of immunoregulatory cells such as M2 macrophages, Th2 cells, myeloid-derived suppressor cells (MDSC), and regulatory T cells (Treg) [32–34]. The interactions of these cells shape the TME, which is crucially important for the treatment of tumors and the prognosis of patients. These studies have significantly contributed to establishing a biological background for our systematic understanding of the TME.

Given this insight into the TME, we employed a top-down approach to systematically dissect the TME, representing the outcome layer, immune role layer, cell layer, cellular component layer, and gene layer. Subsequently, employing a bottom-up approach, we integrated information across various layers and developed a neural network named TME-NET. This model allows us to predict the response to immunotherapy and to conduct a comprehensive assessment of the significance of various cells and key molecules within the TME across pan-cancer cohorts.

## Methods

### Pan-cancer immunotherapy cohorts

To enhance the ability of the TME-NET to predict the efficacy of immunotherapy, we systematically collected data from 11 ICI cohorts. These included five melanoma datasets: Liu2019 [35], Riaz2017 [36], Gide2019 [37], Van Allen2015 [38], and HugoLo2016 [39]; three renal cancer cohorts: IMmotion150 [40], Miao2018 [41], and Choueiri2016 [42]; one gastric cancer cohort: Kim2018 [43]; one urothelial cancer cohort: IMVigor210 [44]; and one glioblastoma cohort: Prins2019 [45]. All transcriptome data were normalized to transcripts per million values to facilitate subsequent analyses and log2 transformation.

### Immune cell categorization and function within the TME

Categorizing cells into immunostimulatory or immunosuppressive groups presents a significant challenge, as there is no existing curated database for this purpose. This necessitates manual collection of information. Our selection is based on two key criteria. First, the cell type must exhibit a significant difference between responder and non-responder for immunotherapy, while maintaining a moderate fraction within the tumor. Second, our findings align with the established consensus in immunoncology research.

We integrated cell deconvolution techniques with immunological insights to determine the roles of various cell types. Using CIBERSORT [46], we evaluated the differences in cell abundances between responders and non-responders. Cells with higher abundances in responders were classified as having immunostimulatory functions, whereas those with lower abundances were considered to potentially support tumor growth or play neutral roles. These analyses were combined with established knowledge in cancer immunology to comprehensively define the roles and types of cells within the TME.

### Cellular component marker gene curation

Marker genes are essential tools for identifying cell types or subtypes. We extracted marker gene information from the Invitrogen Immune Cell Guide [47], which offers comprehensive details on markers for various immune cell types and subtypes. According to this guide, the proteins encoded by these genes are classified into three cellular components: surface, secreted, and intracellular/transcription factors. These markers can be targeted using specific antibodies provided by Invitrogen, and are frequently utilized for cell sorting via flow cytometry. Furthermore, it is essential to convert protein symbols to their corresponding gene symbols.

We focused on eight cell subtypes: M2 macrophages, Tregs, Th2, Th1, DC, NK cells, CD8 effectors, and M1 macrophages. Since the Invitrogen Immune Cell Guide does not provide marker information for MDSC, we consulted the CellMarker and ImmPort databases for this cell subtype. The database offers a range of alternative marker genes for specific cell types or subtypes. We investigated markers identified in MDSC research as described previous in studies [48, 49]. Additionally, we verified their cellular component information using the UniProt database, which contains comprehensive sub-cellular locations of proteins. Additionally, although CellMarker, ImmPort, and UniProt all provide marker genes for various cell types, integrating these three sources ensures consistency. When inconsistencies arise, experimental evidence should take precedence.

### TME-NET architecture design and implementation

Given that the TME influences immunotherapy outcomes, we conceptualized the TME as a hierarchical multi-layer system to simulate how the TME impacts clinical phenotypes. According to this conceptualization, TME-NET is divided into five layers: the gene layer, cellular component layer, cell layer, immune role layer, and output layer. Based on this network architecture, we assigned specific TME components to their respective layers.

To predict two immunotherapy outcomes, responders and non-responders, we utilized a cross-entropy loss function, which is formulated as follows:


$$ \boldsymbol{L}=-\left(\ \boldsymbol{y}\boldsymbol{log}\ \left(\hat{{y}}\right)+\left(\mathbf{1}-\boldsymbol{y}\right)\boldsymbol{\log}\ \left(\mathbf{1}-\hat{{y}}\right)\ \right) $$

where $\mathbf{y}$ is the binary label indicating the outcomes of immunotherapy (**1** for Responder and 0 for non-Responder), and $\hat{{y}}$ represents the predicted probability of immunotherapy response.

To implement our model, we utilized the PyTorch deep neural network framework [50]. The connections between the first three layers—the gene layer to the cellular component layer, the cellular component layer to the cell layer, and the cell layer to the immune role layer—are sparsely connected. Node connections across layers are guided by real biological relationships. Once cell roles and marker genes were confirmed, the node connections were determined. Since the top three layers follow these biological relationships, the connections are sparsely distributed. To manage these sparse connections within our neural network model, we employed a masking matrix *M* alongside the weight matrix *W*. The mask matrix *M*, with elements *M_ij_*, is defined such that *M_ij_* = 1, if the connection between nodes *i* and *j* is active, and *M_ij_* = 0 otherwise. During the training process, we updated the weights by applying the mask to both the weights and their gradients, ensuring that only the weights corresponding to active connections were updated. The weight update rule was formulated as follows: 


$$ \textrm{W}\leftarrow \textrm{W}\odot \textrm{M}-\eta (\nabla \boldsymbol{L} \odot \textrm{M}) $$

Here, $\odot$ denotes the Hadamard product (element-wise multiplication), $\eta$ represents the learning rate, and $\nabla \boldsymbol{L}$ is the gradient of the loss function *L* with respect to the weights. This approach effectively excludes the weights of unconnected edges from the update process, adhering to our model’s design of sparse connectivity. Consequently, we customized the predefined PyTorch modules to accommodate these sparse connections.

### TME-net training and evaluation

Our deep learning network, TME-NET, was trained and evaluated using a dataset comprising 948 patients and 33 848 genes. We partitioned the dataset into a training set and a test set at 7:3 ratio. For training, we selected 138 genes from the original 33 848 genes based on the architecture of the network. The model was trained to perform binary classification using cross-entropy loss. It was benchmarked against other models, all using the same dataset split with a fixed seed. We employed both the Area Under the Curve (AUC) and accuracy as metrics to assess the performance of the model.

### TME-NET ablation study design

To elucidate the influence of different cell types on the performance of our TME-NET model, we plan to conduct an ablation study by systematically removing each of the following cell types: ‘Th1’, ‘Dendritic cell’, ‘CD8 Effector’, ‘M1 macrophage’, ‘NK’, ‘MDSC’, ‘M2 macrophage’, ‘Treg’, and ‘Th2’. Additionally, we isolated related components, including genes and cellular components.

Each ablated variant of the model was compared to a baseline model that incorporated all cell types. Additionally, we analyzed changes in the weights assigned to each cell type and gene within the network to determine their relative importance and influence on model predictions. The ablation study involved training distinct instances of the model with individual cell types removed, adhering to the original data split of 7:3 for training and testing. To ensure the robustness of our results, we repeated this process 100 times using the same dataset. The results were then averaged across multiple runs to confirm statistical robustness.

### Development of the TME-NET designer

To enhance user convenience, we developed a TME-NET designer using advanced technologies. The user interface is powered by Shiny [51], a web application framework for R, with themes customized via the shinydashboard package [52]. Network visualization is facilitated by visNetwork [53]. TME-NET’s code is generated using predefined code templates. Users interact with TME-NET through a three-step process, during which three important neural network weight matrix masks are produced. The three matrices encompass relationships between genes and cellular components, cellular components and cell types, and cell types and their immune roles. We integrate these masks into the predefined code template and package them into a single downloadable zip file for users.

## Results

### Overview of TME dissection and network design

**Figure 1:**
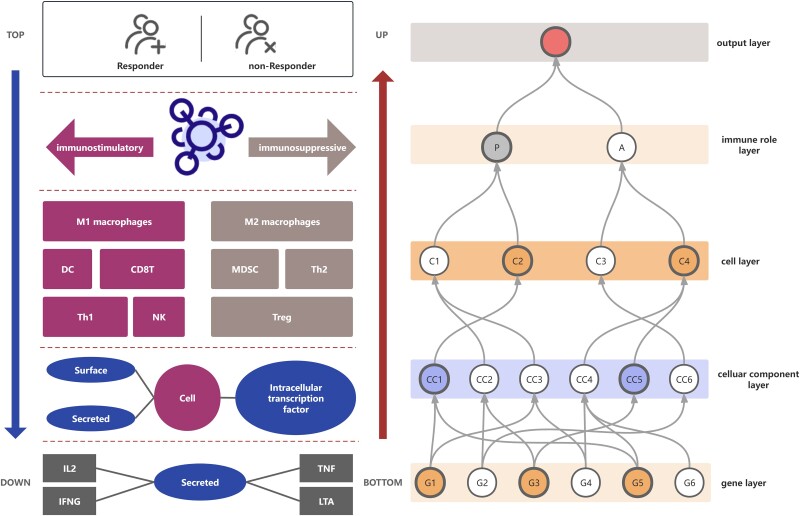
Overview of TME-NET. This schematic illustrates the hierarchical structure of the TME-NET, derived from biological insights. The network is dissected from a top-down perspective into several layers: The output layer, immune role layer, cell layer, cellular component layer, and gene layer. The immune role layer distinguishes between immunostimulatory and immunosuppressive functions. M1 macrophages, DC, CD8+ T cells, Th1 cells, and NK cells play immunostimulatory roles, while M2 macrophages, MDSC, Th2 cells, and Tregs have immunosuppressive functions. Each cell type is further characterized by three cellular components: Surface proteins, secreted factors, and intracellular transcription factors. The gene layer at the bottom of the network specifies the genes associated with each cellular component. To the right, the conceptual model of the TME is depicted, providing a theoretical framework for the network design.

The TME should be viewed as a complex and intricately layered system. To better understand and employ its dynamics, we designed a neural network named TME-NET. This network is structured into five hierarchical layers, with each layer derived from the TME and spanning from the foundational gene layer to the topmost output layer (Fig. 1). This systematic approach involving top-down deconstruction and bottom-up integration of TME components is essential for developing predictive models that are designed to improve immunotherapy outcomes.

**Figure 2:**
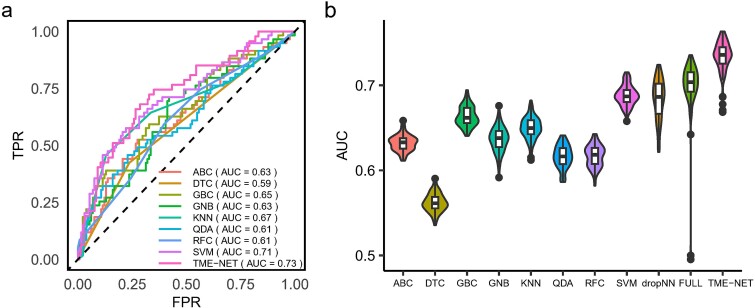
Model benchmarks: (a)the AUCs of KNN, RFC, DTC, GBC, ABC, GNB, QDA, SVM, and TME-NET. (b) the AUC distribution for each model, calculated over 100 training and test runs.

After outlining the general structure and objectives of our neural network model, we assign specific TME components to each layer and establish connections between these components. The output layer is designated for outputting the results of immunotherapy predictions. The immune role layer comprises two principal nodes that represent the immunostimulatory and immunosuppressive functions of various cell types. Specific cell subtypes associated with each role were identified through bulk cellular deconvolution (Supplementary Fig. S1) and existing knowledge of the immune system. Specifically, immunostimulatory functions are supported by five cell types: M1 macrophages, DCs, CD8+ T cells, Th1 cells, and NK cells. Conversely, immunosuppressive functions include four cell types: M2 macrophages, MDSCs, Th2 cells, and Tregs. Together, these cell types comprise the cell layer, which consists of nine nodes. Each of these cell types is characterized by three distinct components, surface proteins, secreted molecules, and transcription factors, culminating in the cellular component layer, which includes 27 nodes, each representing a specific cellular component. At the base of our network, the gene layer, crucial genetic information is represented by 136 nodes, corresponding to 136 genes. All adjacent layers have sparse connections, except for the connection between the immune role layer and the output layer, which is densely connected. Leveraging these architectural specifics, TME-NET incorporates a total of 218 learning parameters, forming an efficient yet sparse network that effectively predicts therapeutic outcomes.

### Immunotherapy response prediction benchmark for multi-models

In order to comprehensively assess the performance of TME-NET, we compared it with a variety of established machine learning models. These included the k-nearest neighbors (KNN), random forest classifier (RFC), decision tree classifier (DTC), gradient boosting classifier (GBC), adaboost classifier (ABC), gaussian naive bayes (GNB), quadratic discriminant analysis (QDA), and support vector machine (SVM). We benchmarked all these models using pan-cancer immunotherapy cohorts that included 948 patients.

According to our comprehensive performance assessment, TME-NET demonstrated superior efficacy, with an AUC value of 0.73 (Fig. 2a). Compared with traditional machine learning models, the SVM model exhibited notable performance, with an AUC of 0.71. Overall, TME-NET outperformed traditional machine learning methods in terms of performance.

To assess the impact of data partitioning on model efficacy, we evaluated the robustness of our model across 100 distinct dataset splits. Moreover, to assess the performance of the neural network, we introduced a fully connected neural network (FULL) and a dropout network with a dropout probability of 0.2 in only the first layer (DropNN), both of which have structures similar to TME-NET. Alongside these deep neural networks, we incorporated a suite of traditional machine learning models, resulting in a comprehensive comparison of 11 different models. Our results demonstrated that TME-NET consistently outperformed other models, achieving higher AUC values (Fig. 2b). Statistical comparisons via t-tests revealed that all comparisons between the TME-NET model and the other models yielded p-values less than 0.001, confirming its superior predictive performance. Notably, all the deep neural network performances surpassed those of traditional machine learning models in terms of predictive accuracy. However, the traditional machine learning models exhibited greater AUC stability than did their deep learning counterparts.

### Assessing cellular and genetic influences in the TME

TME-NET demonstrates remarkable efficacy in predicting immunotherapy outcomes. Distinct from other deep neural networks, TME-NET is meticulously crafted based on the biological intricacies of the TME and immunological mechanisms, with each node representing a specific TME component. Specially, TME-NET integrates a diverse array of cell types and genes, enabling holistic evaluation of their collective impact within a unified computational framework. This comprehensive approach not only facilitates accurate predictions of immunotherapy responses but also generates a detailed set of model weights that reflect varying degrees of influence across different components. Furthermore, acknowledging the potential for variability in training outcomes due to the initial weight parameters of deep learning models [54], we implemented a robust strategy of repeating the training process 100 times [55]. This repetitive training regimen allowed for a thorough analysis, ensuring consistency and reliability in our predictive modeling.

**Figure 3:**
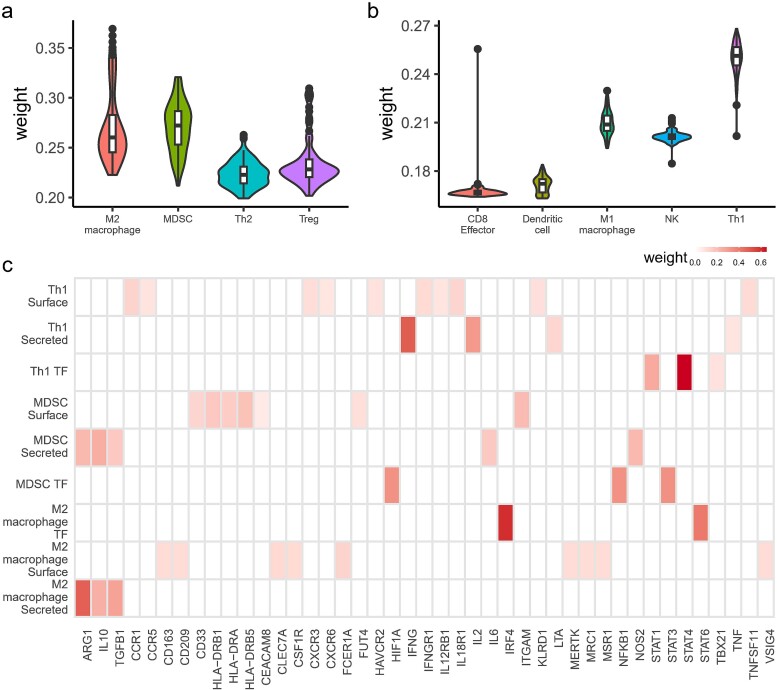
Visualization of TME-NET node weights: (a) weights of immunosuppressive cells, (b) weights of immunostimulatory cells, (c) weights of genes associated with each cellular component. All the results are based on the model being trained and tested 100 times.

In the cell layer of our network, the distribution of weight parameters for immunostimulatory and immunosuppressive cell types was analyzed, as depicted in Fig. 3a-b. The results demonstrate a relatively concentrated distribution of these weights, highlighting their stability. Specifically, within the immunostimulatory group, Th1 was distinguished by a significantly higher weight, with an average value of approximately 0.45, underscoring their dominant role in tumor suppression [56]. M1 macrophages and NK cells also contribute notably, whereas CD8+ effector T cells and DC show comparatively weaker influences. Conversely, among the immunosuppressive cell types, MDSCs exhibit a slightly greater weight than M2 macrophages, indicating a stronger immunosuppressive function [57] (Fig. 3b). Th2 and Treg cells contribute to immunosuppression [58, 59], yet they possess slightly less weights than other cell types.

Visualization through TME-NET has elucidated the pivotal roles played by specific cell types within the TME, with M2 macrophages, MDSCs, and Th1 cells identified as crucial contributors. Furthermore, our analysis revealed that the specific functions of these cells are predominantly dictated by the gene activity associated with secreted proteins and key transcription factors. Prompted by these insights, we sought to identify which genes are critical for maintaining these essential cellular functions. By leveraging our system-wide framework of TME-NET, we were able to pinpoint the key genes that influence the broader network, underscoring their integral roles in the TME.

By employing model weight analysis, we discerned distinct gene influences within various cell types (Fig. 3c). Within Th1 cells, the transcription factor STAT4 was the most influential, as demonstrated by its highest weight [60]. Additionally, the gene IFNG, which encodes gamma interferon, also exhibited considerable weight. Conversely, genes associated with membrane proteins did not display a concentrated weight distribution. In M2 macrophages, the IRF4 gene was identified as particularly crucial [61], alongside the significant weight of the secreted gene ARG1 [62]. In contrast, the weight distribution of MDSCs lacked a similar pattern of concentration. In addition to analyzing the connection weights between adjacent layers, comprehensive analysis of the overall weight distribution of TME-NET as also performed (Supplementary Fig. S2). These findings provide profound insights into the differential roles and impacts of various cells in the TME, enhancing our understanding of their contributions to cellular functions.

Overall, our TME-NET enables the direct visualization of immunostimulatory and immunosuppressive effects of different cells within the TME. Furthermore, it facilitates a detailed examination of the gene influences associated with these cells. This analysis provides critical insights into the components of the TME within a systematic framework, thereby enhancing our understanding of cellular interplay and functions.

### Key genes representing prognostic indicators

Utilizing the TME-NET model within our comprehensive systemic framework has enabled us to intuitively capture and quantify the significant roles of various transcription factors and secreted proteins. These molecules are critical, influencing not only the behavior of individual cells and their immediate neighbors but also playing a pivotal role in the evolution of the TME. Their impact extends to potentially improving patient prognosis by modulating cellular interactions and responses within the TME.

**Figure 4:**
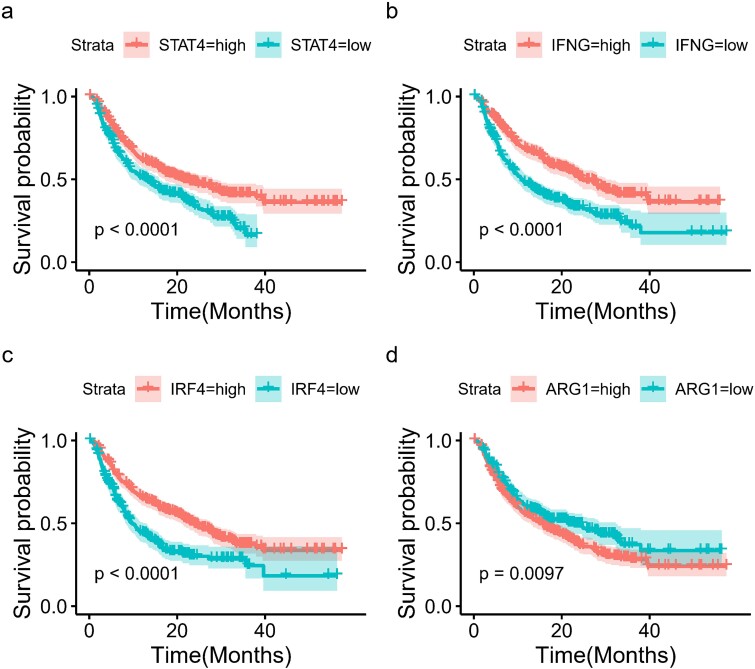
Survival analysis of patients stratified by expression of key genes: (a) STAT4, (b) IFNG, (c) IRF4, and (d) ARG1.

In our detailed analysis, we identified four key genes: STAT4, IFNG, IRF4, and ARG1 as critical players within the TME (Fig. 4a-d). Building on this discovery, we further assessed their potential to predict survival outcomes for patients undergoing immunotherapy. The findings revealed that, with the exception of ARG1, the genes STAT4, IFNG, and IRF4 possess significant predictive capabilities for patient prognosis. Among the four genes, IFNG demonstrated the best performance. A similar conclusion was derived from analyzing the differences in gene expression and classification accuracy, as quantified by the AUC, using these genes (Supplementary Fig. S3). These results not only underscore the pivotal roles of these genes in modulating the TME but also illuminate their prospective clinical implications in enhancing the efficacy of immunotherapy strategies.

### Ablation study for specific cell types

Our analysis provides a clear delineation of the distinct roles that various cell types assume within the TME during immunotherapy. We focused specifically on evaluating the impact of the absence of certain cell subsets on immunotherapy outcomes and on the efficacy of our model.

**Figure 5:**
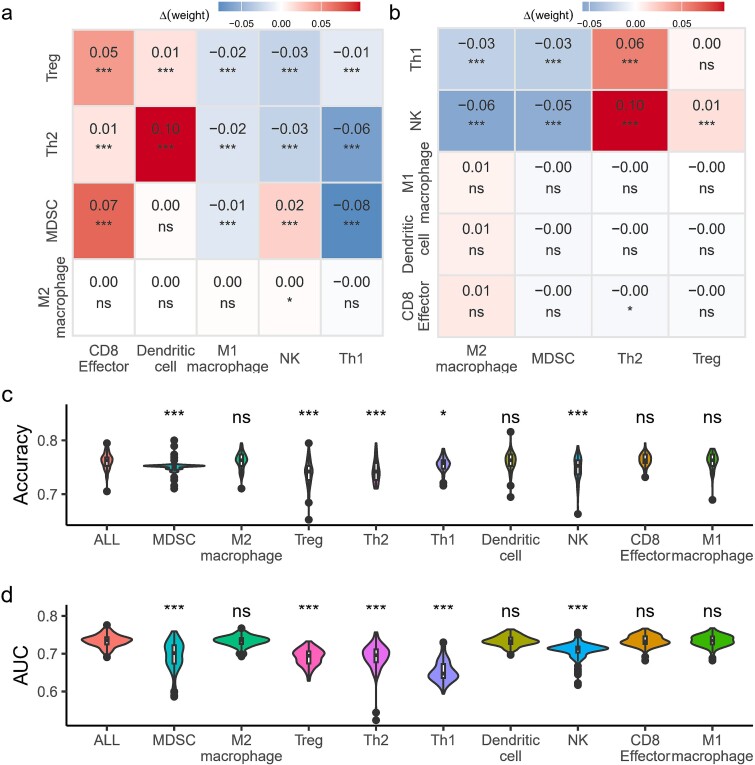
Ablation study for each cell type. (a) Immunosuppressive cell ablation test: This panel demonstrates the effects of ablating each immunosuppressive cell type. Cells are ablated on the left, with their impact on immunostimulatory cells depicted at the bottom. Weight changes are color-coded based on their magnitude, and t-test p-values are provided to quantify significance. (b) Immunostimulatory cell ablation test: The consequences of ablating each immunostimulatory cell are shown. The cells in this group are listed on the left, and the immunosuppressive cells are displayed at the bottom. Weight changes are color-coded, with p-values provided similarly to (a). (c) Accuracy distribution: This panel presents the model’s accuracy distribution when specific cells are removed, labeled at the bottom. ‘All’ denotes the scenario where no cells have been removed. (d) AUC distribution: Echoing panel (c), this panel outlines the AUC distribution under conditions where individual cells are removed, with ‘all’ signifying the inclusion of all cells.

Our ablation experiments demonstrated variable effects on weight changes, which differed significantly across various cell types. We assessed the cell types from an immunostimulatory role perspective. The absence of Th1 or NK cells significantly influenced the weights of immunosuppressive cells (Fig. 5b). However, when any of the following cells—Dendritic cells, NK cells, CD8+ Effector cells, or M1 macrophages—are absent, the weights of the immunosuppressive cell types remain unchanged. Conversely, from the perspective of immunosuppressive roles, the exclusion of M2 macrophages had no effect, whereas the absence of any of the following cells—MDSCs, Tregs, or Th2 cells—led to significant changes in the weights of immunostimulatory cells (Fig. 5a). We have detailed the cellular weights and model performance from the cellular ablation experiments in the supplementary files (Supplementary Tables S1 and S2). These findings suggest that the regulatory interactions between immunostimulatory and immunosuppressive cell types are complex and cell-type specific, influencing weight changes in a context-dependent manner.

Our ablation experiments revealed heterogeneous effects on model performance, with variations distinctly associated with specific cell types. Notably, the removal of any of the following cell types—MDSCs, Tregs, Th2 cells, or NK cells—resulted in a significant decrease in model accuracy (Fig. 5c). Furthermore, the absence of any of the following cell types—MDSCs, Tregs, Th2 cells, Th1 cells, or NK cells—led to a substantial reduction in the AUC, with Th1 cells showing the most pronounced impact (Fig. 5d). These findings emphasize the critical role of these cell types in maintaining the predictive reliability and overall efficacy of our model.

### Online TME-NET designer

The TME plays a pivotal role in shaping clinical outcomes across a range of medical contexts, extending well beyond its established impact on immunotherapy responses. To address this critical influence, we introduced the TME-NET designer (https://immbal.shinyapps.io/TME-NET/), a versatile online tool designed to expand the practical applications of TME insights. This platform enables researchers not only to predict diverse clinical outcomes but also to gain a deeper understanding of TME dynamics. Additionally, no data is retained after the user completes the customized TME-NET.

**Figure 6:**
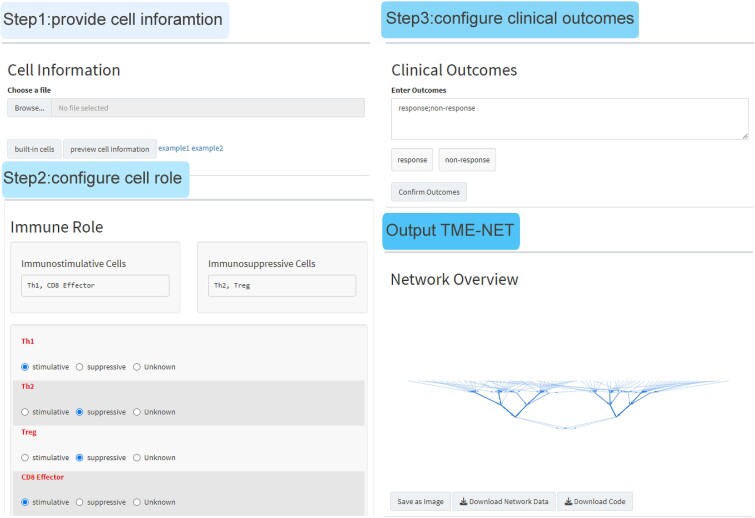
Operational workflow of the TME-NET designer. Step 1: Users may upload their tailored cellular information or opt for the built-in cell data. Step 2: Immune cells are assigned to each cell type by the user. Step 3: Users are required to input their clinical outcomes of interest. Final output: A customized TME-NET, including its structure and corresponding python code, is generated for download.

Users are prompted to input three essential types of biological data: details on immune cells, their functional roles within the TME, and the clinical outcomes of interest (Fig. 6). Here, to facilitate the configuration of cell information, we provide built-in cells for reference or direct use. The detailed file formats can be found in the supplementary files (Supplementary Table S3). After data input, the tool processes the information to dynamically generate a tailored TME-NET model. This model incorporates the specific data provided, reflecting the nuanced interplay within the TME. The output includes not only the model’s structure but also the executable code necessary for its implementation, allowing for further analytical explorations and applications in TME research. Currently, TME-NET only provides online network design and code generation with real-time functional updates. However, it does not include model training. Given the vital role of the TME in broader tumor phenotypes, TME-NET can be applied to various other clinical settings with the assistance of the TME-NET designer.

## Discussion

Guided by the biological implications and immune mechanisms within the TME, we performed a methodological synthesis of top-down deconstruction and bottom-up integration. This approach was instrumental in elucidating the complex information flow characteristics of the TME. We designed a deep neural network TME-NET characterized by sparse connections that reflect intricate component connections within the TME. Using this model, we achieved not only more efficacious predictions of immunotherapy outcomes but also a detailed examination of key cellular and genetic elements within the TME. These insights hold significant potential to deepen our understanding of immunotherapy mechanisms and to inform the development of more targeted therapeutic strategies.

To enhance the interpretability of our model, the structure of the TME-NET was meticulously aligned with biological implications. Each node within the network corresponds directly to a specific gene or biological entity, and every connection accurately reflects the hierarchy of these biological entities. TME-NET integrates diverse cellular and genetic information across multiple scales, organizing them into distinct layers from immunotherapy outcomes down to the gene level. This structure provides comprehensive, multidimensional insights into the TME. Comparable models, such as P-NET [26] and PSA-NET [27], have been designed around curated pathways, incorporating primarily gene and pathway information. Similarly, the Dcell [28] and DrugCell [29] networks utilize curated Gene Ontology (GO) [63] terms to furnish detailed gene annotations. Models guided by biological knowledge deliver exceptional performance; however, their interpretability primarily offers insights into pathways or functions. Researchers increasingly favor a richer dimension of interpretability that encompasses more comprehensive aspects. The TME-NET model incorporates a broader range of scale information. An information chain across multiple scales provides a more integrated insight from clinical outcomes, cells, and genes, enhancing its interpretability.

Benefiting from its refined structural design, TME-NET provides a robust computational framework that facilitates the independent analysis of individual components within the TME. This analysis significantly advances our understanding of the TME, thereby informing the development of immunotherapy strategies. Through visualization of cellular weights and targeted ablation studies, Th1 cells have been identified as pivotal for the efficacy of immunotherapies. Further evidence from TME-NET weight visualization underscores the critical role of IFN-γ, which is secreted by Th1 cells, in modulating immune responses. Specifically, Th1 cells are instrumental in enhancing the therapeutic effects of PD-1 blockade therapies by fostering an IFN-γ-mediated immune response across various cancers [64–66]. IFN-γ production by Th1 cells is crucial for the activation and proliferation of CD8+ cytotoxic T cells, which are capable of recognizing and eliminating cancer cells [67, 68]. Moreover, IFN-γ enhances the capacity of DC to present antigens, thereby improving T-cell recognition of cancer cells [69, 70]. Although these findings delineate the mechanistic contributions of Th1 cells to the immune process, they do not resolve the broader systemic question of which component is the most critical from a systemic perspective in the overall immune response, a determination that is crucial for refining immunotherapy strategies.

TME-NET is an open research framework designed to be interactive and initially user-friendly. Researchers can engage with the model through the online TME-NET designer, allowing them to customize models with fewer parameters for their specific clinical tasks in real time. Although established models such as P-NET and Dcell offer customized solutions based on established biological networks, they also face significant challenges. While the network aims to provide no customization flexibility, it is designed using comprehensive biological knowledge sourced from databases such as Reactome and Gene Ontology (GO). In this context, the network often has a vast number of parameters. This makes them particularly difficult to tune, especially for smaller sample sizes, and thus not very user-friendly. However, configuring cell information presents its own set of challenges. Users are required to specify the cell type of interest, marker genes for cellular components, and immune role of each cell type. Fortunately, specific cell types can be identified using single-cell atlases such as the Single Cell Expression Atlas [71, 72], TISCH [73, 74], and the Single Cell Portal [75], which facilitate the analysis of tumor tissues. Marker genes are readily available from databases such as Immport [76], CellMarker [77], and UniProt [78], which provide comprehensive and detailed annotations for these biomarkers. The immune role is determined through cellular deconvolution of bulk RNA-seq data, where differences between phenotypes help indicate the immune role. This detailed information is often further validated by extensive literature.

To balance accuracy with interpretability, we developed the TME framework. Constructed from a series of biological entities, this framework simulates information flow across multiple biological scales, offering enhanced accuracy and deeper biological insights into the TME. As more precise cell type information is incorporated, the TME-NET’s potential for accuracy and interpretability increases. Meanwhile, given that the TME determines various clinical phenotypes, any cell of interest for a specific clinical phenotype would be included to customize the TME-NET. Within this framework, interpretable visualization will be provided to explore the influences of cells or genes.

## Conclusions

To address the challenge of predicting immune responses in immunotherapy, our research concentrated on the TME from a holistic perspective. We have established a conceptual layered system within this context. Based on this framework, we developed and implemented a deep neural network that outperforms traditional machine learning models in terms of efficacy. By visualizing the network nodes, we were able to identify key cells and genes within a simulated system, thus enhancing our understanding of the TME in a structured manner. This study not only shows substantial clinical utility but also provides a detailed framework for enhancing our systematic comprehension of immunotherapy.

**Key Points**

- A multi-layered conceptual model of the tumor microenvironment (TME), characterizing it across multiple scales, was proposed using a top-down approach.
- TME-NET is a deep neural network designed for predicting pan-cancer immune checkpoint inhibitor responses, providing high predictive performance and enhanced interpretability for the TME.
- A renewed emphasis on the role of Th1 cells within the TME has been prompted by TME-NET’s visualization analysis; specifically, the cytokine-encoding gene IFNG from Th1 cells was identified as a key prognostic factor for immunotherapy.
- An online designer for customized TME-NET can help researchers extend TME-NET applications across various clinical settings.

## Supplementary Material

Supplementary_figures_bbae410

Supplementary_tables_bbae410

## Data Availability

All data utilized in this study are publicly available, as detailed in the Methods section. The paper provides web links or unique identifiers for the referenced public cohorts/datasets. The online database used was https://immbal.shinyapps.io/TME-NET.
