# Supplementary material for: TME-NET: an interpretable deep neural network for predicting pan-cancer immune checkpoint inhibitor responses
Source: Brief Bioinform. 2024 Aug 21;25(5):bbae410. doi: 10.1093/bib/bbae410 (PMC11337220; doi:10.1093/bib/bbae410)
Supplement: Supplementary_figures_bbae410 [file supplementary_figures_bbae410.docx]

**TME-NET: An interpretable deep neural network for predicting pan-cancer immune checkpoint inhibitor responses**

Xiaobao Ding^1,2^, Lin Zhang^1^, Ming Fan^1*^, Lihua Li^1*^

^1^Institute of Biomedical Engineering and Instrumentation, Hangzhou Dianzi University,

310018, Hangzhou, Zhejiang, China

^2^Institute of Big Data and Artificial Intelligence in Medicine, School of Electronics and Information Engineering, Taizhou University, 318000, Taizhou, Zhejiang, China

* Corresponding author

*Ming Fan - Email: [ming.fan@hdu.edu.cn]

*Lihua Li - Email: [lilh@hdu.edu.cn]

**
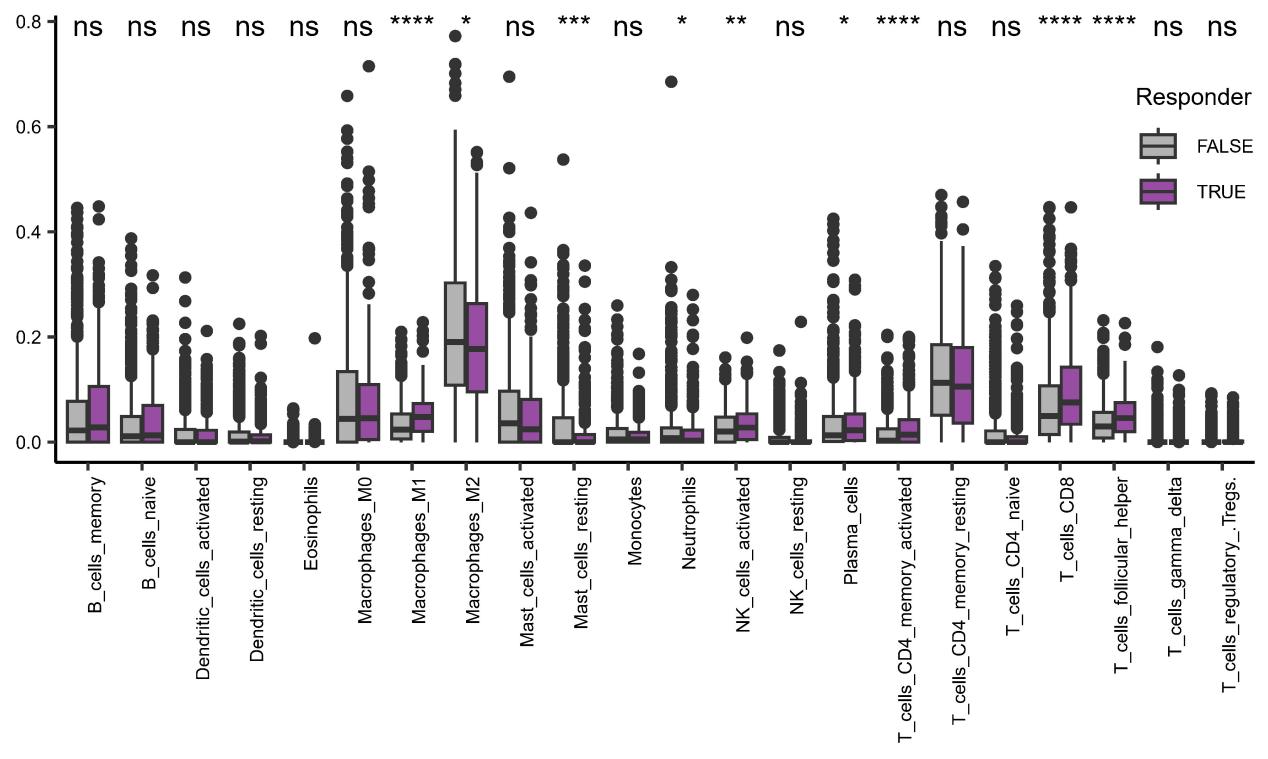
**

**Figure S1:** Cellular deconvolution of the immunotherapy cohort. The figure demonstrates variations in cell abundance between responders (purple) and non-responders (gray). Differences between the two groups were statistically analyzed using a t-test.

**
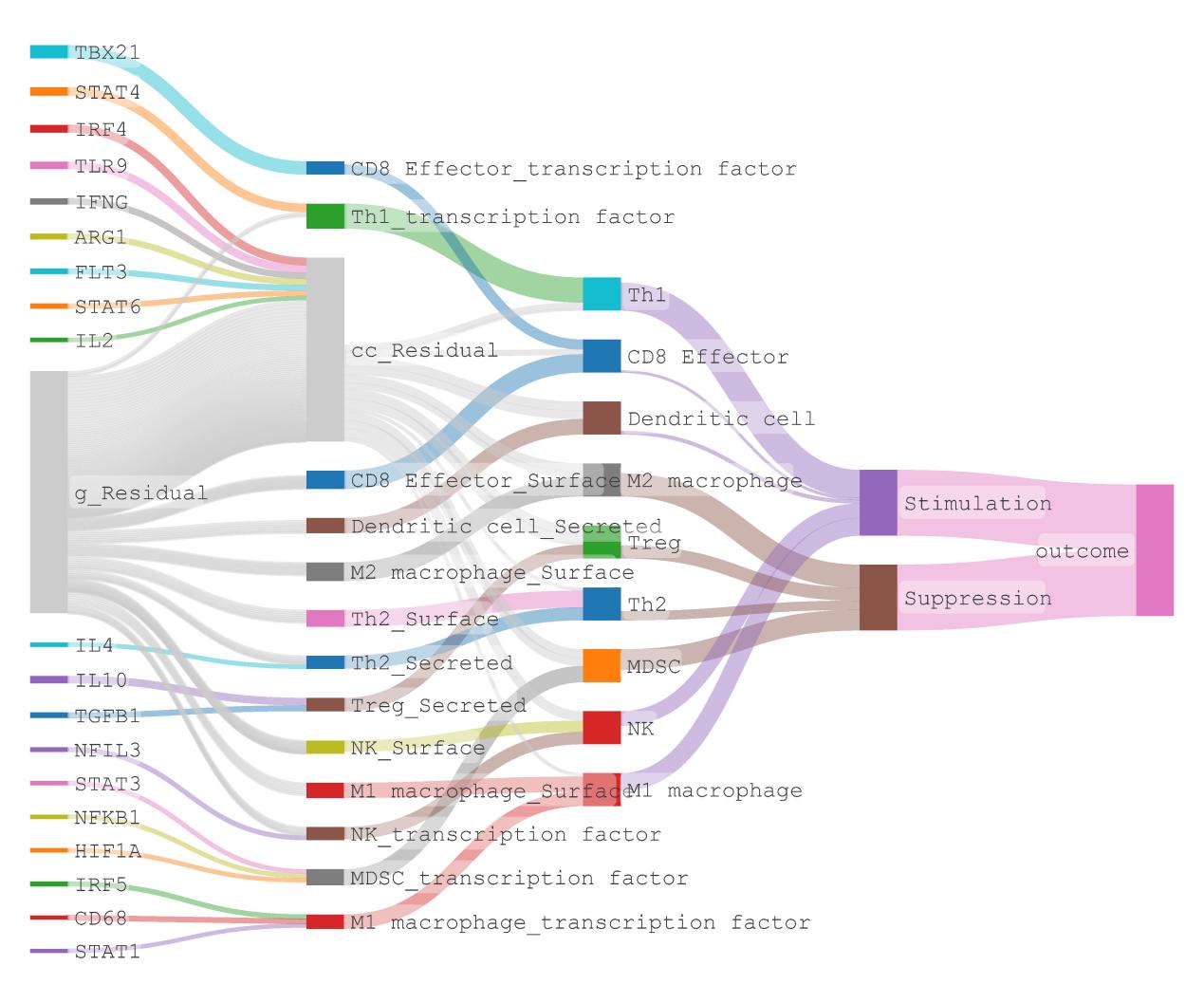
**

**Figure S2:** Overview of model weight distribution. Line thickness represents the connection weight. For clarity, smaller connection weights (less than 0.3) are aggregated into 'g_Residual' and 'cc_Residual' categories.

**
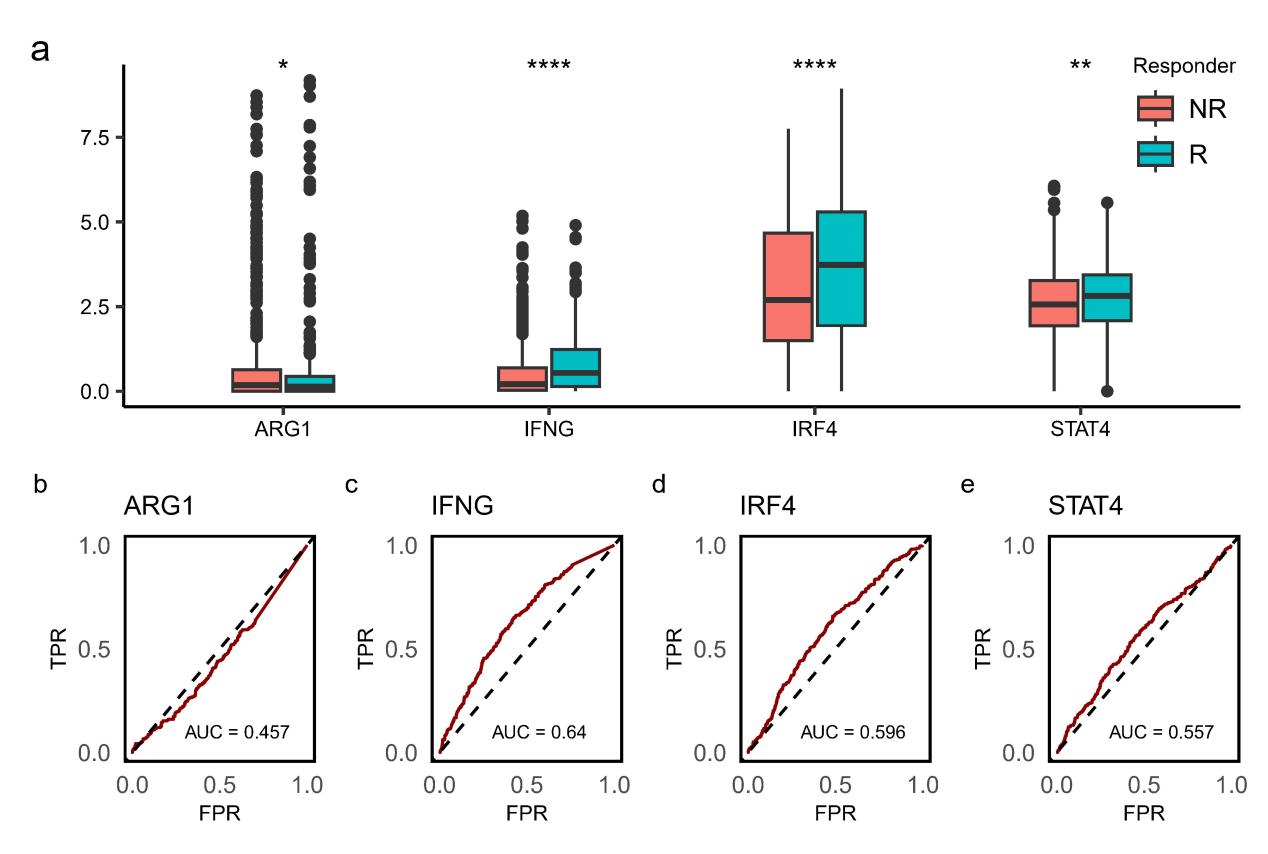
**

**Figure S3:** Overview of the four key genes within the immunotherapy cohort. Panel (a) displays the differences in gene expression between non-responders (NR) and responders (R). Panel (b) illustrates the classification performance of these four genes within the immune cohort.
